# Plasmon-coupled Charge Transfer in FSZA Core-shell Microspheres with High SERS Activity and Pesticide Detection

**DOI:** 10.1038/s41598-019-50374-y

**Published:** 2019-09-25

**Authors:** Donglai Han, Jiacheng Yao, Yingnan Quan, Ming Gao, Jinghai Yang

**Affiliations:** 1 School of Materials Science and Engineering, Changchun University of Science and Technology, Changchun, 130022 P.R. China; 2 Key Laboratory of Functional Materials Physics and Chemistry of the Ministry of Education, Jilin Normal University, Changchun, 130103 P. R. China; 3 National Demonstration Centre for Experimental Physics Education, Jilin Normal University, Siping, 136000 P. R. China; 4 Key Laboratory of Preparation and Application of Environmental Friendly Materials, Jilin Normal University, Ministry of Education, Changchun, 130103 P. R. China

**Keywords:** Raman spectroscopy, Nanosensors

## Abstract

A commercial SERS substrate does not only require strong enhancement, but also can be reused and recycled in actual application. Herein, Fe_3_O_4_/SiO_2_/ZnO/Ag (FSZA) have been synthesised, which consisted of Fe_3_O_4_ core with strong magnetic field response and an intermediate SiO_2_ layer as an electronic barrier to keep the stability of magnetite particles and outer ZnO and Ag as the effective layers for detecting pollutants. The SERS enhancement factor (EF) of the FSZA was ~8.2 × 10^5^. The enhancement mechanism of the FSZA core-shell microspheres were anatomized. The electromagnetic enhancement of surface deposited Ag, charge transfer, and molecular and exciton resonances act together to cause such high enhancement factors. For practical application, the FSZA core-shell microspheres were also used to detect thiram, moreover, which was collected and separated by an external magnetic field, and maintained the SERS activity without significant decline during multiple tests. So the good enhancement performance and magnetic recyclability make the FSZA core-shell microspheres a promising candidates for practical SERS detection applications.

## Introduction

Surface-enhanced Raman scattering (SERS) is a strong spectroscopy technology with high sensitivity and fast response. It is widely used in chemical, pharmaceutical, biosensing, food safety and environmental monitoring^1–4^. The substrate materials play a key role in SERS enhancement effect, because the substrate material can affect the enhancement effect^5^. Traditional novel metal substrates have high SERS activity but are too technologically demanding and expensive^6^. In contrast, semiconductor SERS substrates not only have higher chemical stability but also have better biocompatibility^7^. So people turn their attention to the semiconductor SERS substrate due to their better chemical stability and biocompatibility. Especially, ZnO has excellent properties in supporting chemical enhancement^8^. However, the SERS activity of pure ZnO is too weak to be practically applied. This results in enhanced SERS effects by manipulating the heterostructure between the noble metal and ZnO. It is widely known that Ag has a higher surface plasma efficiency than Au and is much cheaper than Au. Therefore, a large number of studies on ZnO and Ag heterojunction have emerged, and the SERS intensity of ZnO has been greatly improved^9^. However, the SERS substrate does not only require strong enhancement, but also can be reused and recycled in actual application^10^. Magnetic particles as an important family of separable materials, which have attracted much attention due to their unique separability, enabling them to facilitate the convenient recovery of SERS substrates^11^. However, magnetite particles are instability in harsh environments, especially under acidic conditions^12–14^. So it is very necessary to coat a silica shell on it.

On above mentioned reasons, we prepared Fe_3_O_4_/SiO_2_/ZnO/Ag (FSZA) as a novel SERS substrate. The separability imparted to the substrate by the magnetic core Fe_3_O_4_ is convenient for reuse and recovery, and the surface is covered with an inert SiO_2_ layer to enhance the stability of Fe_3_O_4_. The ZnO/Ag heterostructure further covered thereon combines the advantages of semiconductor and noble metal, imparts higher SERS activity to the substrate and allows for higher SERS uniformity and better chemical stability. The FSZA core-shell microspheres could be further extended to identify thiram or other pesticides and is expected to be an alternative method for rapid and accurate detection of contaminants in food.

## Results and Discussion

### Characterization of the FSZA core-shell microspheres

**Figure 1:**
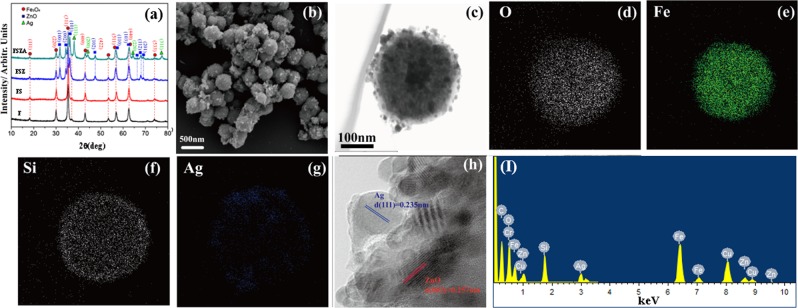
(**a**) XRD patterns of Fe_3_O_4_ (F), Fe_3_O_4_@SiO_2_ (FS), Fe_3_O_4_@SiO_2_@ZnO (FSZ), and Fe_3_O_4_@SiO_2_@ZnO@Ag (FSZA); (**b**) SEM images of FSZA; (**c**) TEM images of FSZA; (**d**–**g**) The elemental mapping of O, Fe, Si, Zn, and Ag of FSZA, respectively; (**h**)HRTEM image of FSZA; (**I**) TEM-SAD image of FSZA.

**Figure 2:**
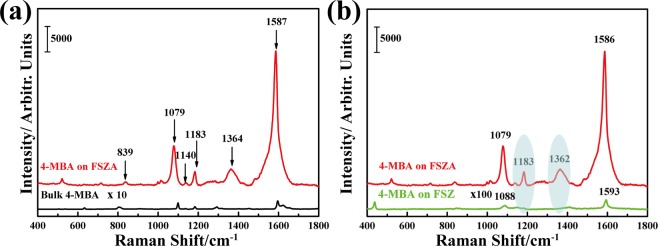
(**a**) Raman spectra of 4-MBA (10^−3^ M) adsorbed on the FSZA core-shell microspheres and 4-MBA powder; (**b**) SERS spectra of 4-MBA molecules adsorbed on FSZ and FSZA.

All prepared products were tested by XRD to confirm crystal structure and compositional changes. The characteristic diffraction peaks labeled (220), (311), (400), (422), (511), (440), and (533) can be indexed as a typical cubic phase of Fe_3_O_4_ (JCPDS card No. 19-0629). The amorphous SiO_2_ coating has no effect on the structure of Fe_3_O_4_. According to the standard JCPDS (No. 36-1451), the main added diffraction peaks at 31.96, 34.64, 36.46, 47.72 and 56.76 can be easily indexed to the hexagonal zinc oxide structure^15^. Compared with Fe_3_O_4_ (F), Fe_3_O_4_@SiO_2_ (FS), and Fe_3_O_4_@SiO_2_@ZnO (FSZ) besides the peaks of Fe_3_O_4_ and ZnO, the apparent characteristic diffractions at 38.11◦, 44.30◦, 64.41◦, and 77.49◦ can be indexed to cubic phase Ag (JCPDS No. 04-0783). It indicates that Ag have been loaded on the surface of core-shell microspheres^16^. Figure 1b shows the SEM image of FSZA, these microspheres are quite uniform and the diameter is about ~220 nm. The TEM images (Fig. 1c) further confirmed the core-shell structure. Figure 1d–g show the distribution of elements O, Si, Fe, Zn and Ag in different colors, which indicated that SiO_2_, ZnO and Ag exist on the outer surface of Fe_3_O_4_ microspheres. Figure 2h shows two different lattice fringes. The fringe distance d = 0.235 nm, which is close to the lattice fringes of (111) plane of Ag (JCPDS card No. 04-0783). One corresponds to the lattice fringe of the Ag (111) plane, d = 0.235 nm. The other one is close to the (002) plane of ZnO, d = 0.257 nm^17^. The results show that a heterostructure of zinc oxide and Ag is formed on the surface of the microspheres. Figure 2I confirms the presence of O, Si, Fe, and Ag elements consistent with XRD and TEM observations. In summary, Ag and ZnO have been immobilized on the surface of the microspheres.

### Evaluating SERS activity of the FSZA core-shell microspheres

Figure 2a shows the Raman spectra of 4-MBA (10^−3^ M) adsorbed on the FSZA core-shell microspheres and 4-MBA powder. The SERS signals at 839, 1079, 1140, 1183, 1364, and 1587 cm^−1^ are observed on the FSZA core-shell microspheres^18,19^. The two dominant peaks at 1079 and 1587 cm^−1^ are assigned to the ring-breathing modes. The Raman band at 839 cm^−1^ is attributed to the COO^−^ bending mode (δ(COO^-^)) and that at 1140 cm^−1^ is attributed to a mixed mode (13β(CCC) + ʋ(C–S) + ʋ(C–COOH))^20^. In addition, the band at approximately 1183 cm^−1^ is mainly due to the a_1_ vibration of v (CH), the band centered at 1364 cm^−1^ is due to the symmetric stretch of the carboxylate group ν(COO^−^). Moreover, the most significant difference is the large increase in Raman signal and peak position movement for the FSZA core-shell microspheres, which is different from the Raman spectrum of 4-MBA powder. These changes may be caused by the interface between ZnO and Ag. All of this evidence suggests that the surface has been significantly enhanced.

The SERS enhancement factor (EF) of the FSZA core-shell microspheres were calculated by the following equation according to previous reports^21,22^:

1 $${\rm{EF}}={{\rm{I}}}_{{\rm{SERS}}}/{{\rm{I}}}_{{\rm{bulk}}}\times {{\rm{N}}}_{{\rm{bulk}}}/{{\rm{N}}}_{{\rm{SERS}}}$$

where N_SERS_ and N_bulk_ are the number of molecules; I_SERS_ and I_bulk_ are the peak intensities. In this case, the determination of EF requires the measurement under the same conditions. The EF for 4-MBA adsorbed on the FSZA core-shell microspheres were calculated through selecting the Raman peak at 1587 cm^−1^. N_SERS_ and N_bulk_ represent the numbers of 4-MBA molecule adsorbed on the FSZA core-shell microspheres and bulk molecule excited by the 514 nm laser beam. For the optical configuration and microscope used in this study, the confocal depth was 21 µm, the laser beam spot diameter was 1 µm, and the 4-MBA density was 1.346 g/cm^3^. Therefore, N_bulk_ is estimated to be 3.3 × 10^10^ molecules in the the detected 4-MBA solid sample region^23^. N_SERS_ is the number of surface adsorbed molecules in the laser spot that can be obtained according to the method proposed by Orendorff *et al*.^24^.

2 $${N}_{SERS}=\frac{{N}_{d}{A}_{laser}{A}_{N}}{\sigma }$$

Where N_d_ is the density of the Ag, A_laser_ is the area of the laser focus, A_N_ is the footprint area of Ag, and σ is the surface area occupied by an adsorbed molecule. A_laser_ can be obtained from the diameter of the laser spot and can be adopted as 0.20 nm^2^/molecule^25^. Therefore, the EF of the FSZA core-shell microspheres was evaluated to be ~8.2 × 10^5^.

### Mechanism of SERS detection

**Figure 3:**
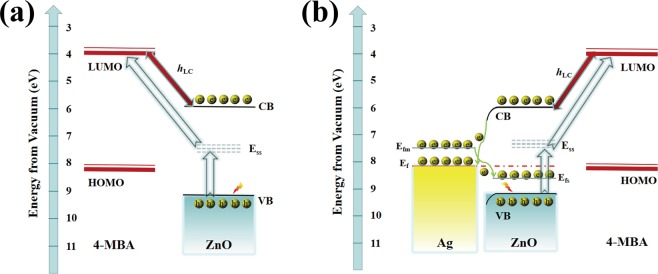
The mode of SERS enhancement mechanism of 4-MBA on (**a**) the FSZ; (**b**) the FSZA core-shell microspheres.

Usually electromagnetic (EM) and charge transfer (CT) mechanisms are the two main principles that contribute to the enhancement of SERS. To determine the appropriate mechanism for the FSZA core-shell microspheres, we compared the SERS activities among the FSZA substrates and the FSZ substrates after being immersed in 10^−3^ M 4-MBA solution in Fig. 2b. Due to the SERS spectra of the FSZ was too weak to be seen when the FSZ and the FSAZ were put together, so we enlarged the SERS intensity of the FSZ by 100 times to see the changes of peaks more clearly. Clearly, FSZA can generate a stronger SERS signal than the FSZ. And the SERS intensities of 4-MBA at 1587 cm^−1^ collected on the FSZA core-shell microspheres substrates are about 2100 times higher than that of the bare ZnO. On the FSZ sample, the main bands of 4-MBA are located at 1593 and 1088 cm^−1^, which are assigned to the v_8a_ (a_1_) and v_12_ (a_1_) aromatic ring characteristic vibrations, respectively. These bands are the main contribution of ZnO-to-molecule CT mechanism. Figure 3a shows the charge transfer process. The photoexcited electrons can be injected into the conduction band (CB) of the zinc oxide and/or then relaxed to the surface level of the zinc oxide (E_SS_) and then transferred to the lowest unoccupied molecular orbital (LUMO) of the molecule adsorbed on the zinc oxide. According to the Herzberg-Teller selection rules^26^:

3 $$\Gamma ({Q}_{k})=\Gamma ({\mu }_{CT})\times \Gamma ({\mu }_{ex})$$

where Γ(μ_CT_) is an irreducible representation of the charge transfer transition, and Γ (μ_ex_) is the exciton transition from which intensity is borrowed. Since the optical transition in 4-MBA is in the ultraviolet, it is likely that intensity for this transition is obtained by borrowing exciton transitions at or near the laser excitation (2.54 eV). So we obtain resonance transitions from the molecule LUMO to the edge of ZnO conduction band (*h*_LC_).

In addition, for the FSZA core-shell microspheres substrates, there are two new bonds appeared at ~1362 and near 1183 cm^−1^ as shown in Fig. 2b. In addtion, the SERS peak of 4-MBA at about 1593 cm^−1^ on the FSZ shifts to 1578 cm^−1^ affter adding Ag. Since the work function of Ag (4.3 eV vs. NHE) is lower than the work function of ZnO (5.2 eV vs. NHE), the Fermi level of Ag is larger than that of ZnO^27,28^. When Ag is sputtered onto the surface of the FSZ, the conduction band of Ag electrons are easier transfer to that of ZnO until their level of Fermi energy attains equilibration. The Fermi level of zinc oxide in the composite material is higher, and the Fermi level of silver is lower. As a consequence of charge redistribution, the SERS activity of the FSZA substrates are higher than that of the FSZ substrates, indicating that the charge transfer from Ag to ZnO leads to the formation of an inter electric field^29,30^. As shown in Fig. 3b, there are three reasons for enhancement, namely, the EM enhancement of Ag, molecular and exciton resonances, and charge transfer. They appear in the form of multiplications, so when they coincide, a large enhancement factor is produced^31,32^.

### Evaluating the SERS activity of the FSZA substrates

**Figure 4:**
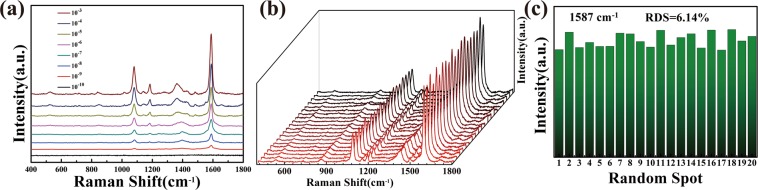
(**a**) SERS spectra of 4-MBA based on FSZA with concentrations ranging from 10^–3^ to 10^–10^ M; (**b**) the 20 SERS spectra of 4-MBA molecules were collected on FSZA substrate; (**c**) SERS intensity of 4-MBA a t 1587 cm^−1^ of the 20 SERS spectra.

Figure 4a shows the SERS signals of different concentrations of 4-MBA, and the intensity of each peak increased with increasing concentration. The detection limit of 4-MBA was 10^−9^ M. Figure 4b shows SERS spectra obtained from 20 randomly selected positions on the FSZA. The calculated relative standard deviation (RSD) of the intensities of the peaks at 1587 cm^−1^ was 6.14%, indicating great reproducibility of the FSZA.

### Reusability of the FSZA core-shell microspheres substrates and detection of thiram

**Figure 5:**
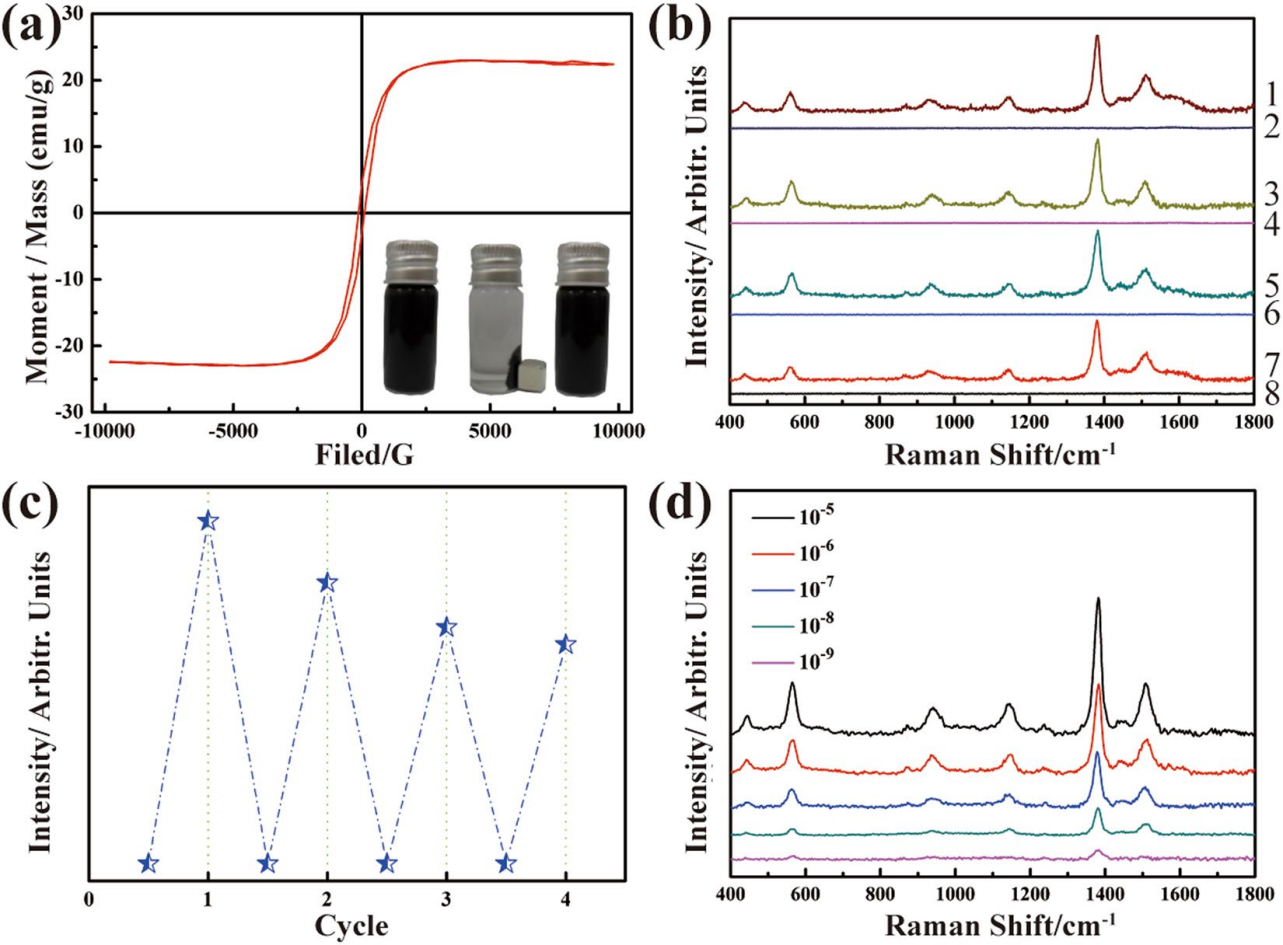
(**a**) Room temperature magnetic hysteresis curves of the FSZA core-shell microspheres, the inset showed the water dispersibility and magnetic separation of the FSZA core-shell microspheres; (**b**) SERS spectra of thiram before and after self-cleaning test; (**c**) corresponding normalized Raman intensities of 1381 cm^−1^ when the SERS substrate is recycling for four times in the detection of 10^–5^ thiram; (**d**) SERS spectra of thiram based on FSZA with concentrations ranging from 10^–5^ to 10^–9^ M.

The magnetic properties of the FSZA core-shell microspheres were shown in Fig. 5a. The samples showed superparamagnetic properties with the saturated magnetization value is 22.7 emu/g. As shown in the inset of Fig. 5a, the uniform dispersion of FSZA can be quickly separated from the solution with an external magnet, and form aggregates in only 22 s. At the same time as the magnet was removed, the aggregate was quickly redispersed into the solution by a slight shaking. Superparamagnetic properties are extremely important for reusable SERS substrates.

The FSZA core-shell microspheres were used as a SERS substrate for further detection of the dithiocarbamate fungicide (thiram), which is widely used in food crops. But rolonged or repeated exposure may result in sensitive skin and have bad effects on the thyroid or liver. To test the reusability of the FSZA core-shell microspheres, we repeat the SERS experiments with the same sample for four times, using a magnet to separate the FSZA from solution after each test. We repeated four times of SERS experiment for the same sample to test the reusability of FSZA core-shell microspheres. Figure 5b shows that the substrate can be regenerated. The SERS signals of thiram almost completely disappeared after visible light irradiation and rinsing. In Fig. 5c, the FSZA core-shell microspheres substrates retained SERS activity completely after four replicates. Although the average Raman intensity is slightly reduced due to the decrease of hot spots and adsorption capacity, it can still meet the testing requirements of thiram. Figure 5d shows the SERS signals of different concentrations of thiram. The Raman peaks of thiram can be detected even at 10^−9^ M (0.0003 ppm), and this concentration is much lower than the 7 ppm maximal residue limit (MRL) in fruit prescribed by the U.S. Environmental Protection Agency (EPA) and may be applied in pesticide residue practical detection. So the FSZA core-shell microspheres could be an efficient SERS substrate in detection of trace thiram in practical detection.

## Experimental

### FSZA core-shell microspheres preparation

Firstly, Fe_3_O_4_ sample was prepared via solvothermal method^33^. 3.24 g FeCl_3_·6H_2_O, 5.4 g C_2_H_3_O_2_Na and 0.003 g C_12_H_25_SO_4_Na were dissolved in 60 mL (CH_2_OH)_2_, stirred and then transferred to a autoclave to get the Fe_3_O_4_. SiO_2_ was coated on Fe_3_O_4_ core through the modified Stöber method^34^. 0.3 g Fe_3_O_4_ was added into a mixed solution of ammonia (3.6 mL), alcohol (120 mL) and deionized water (30 mL) and stirred for half an hour, then continue added 1.05 mL TEOS and stirred for 4 hours. 0.5 mmol ZnNO_3_·6H_2_O, 10 mL DMF, 2 mL NaOH (0.5 mol/L), 30 mL deionized water and 0.24 g Fe_3_O_4_@SiO_2_ were mixed together and manner to get Fe_3_O_4_@SiO_2_@ZnO. For detailed parameters, please see our previous papers^35^. Finally, we used the wet chemistry method to synthesized FSZA core-shell microspheres.

### Characterization

We evaluated the structural of FSZA core-shell microspheresg with X-ray diffraction (XRD, MXP18). The morphology were studied by field emission scanning electron microscope (FE-SEM, JSM-6700F). Raman spectra were recorded on a Renishaw Micro-Raman spectrometer.

### Detection of thiram using FSZA core-shell microspheres

First, thiram was dissolved in ethanol and diluted to a predetermined concentration with water. A certain amount of the FSZA core-shell microspheres were add into the thiram solution, then the mixed solution stirred and washed by deionized water and ethanol before testing, and placed on the substrate for measurement at last.

## Conclusion

In summary, the FSZA core-shell microspheres substrates is designed and prepared by an easy and low cost method. The as-fabricated substrate is ultrasensitive and reproducible.The results demonstrate that this FSZA core-shell microspheres substrates are not only beneficial for the detection of pesticides but also has a good application prospect for SERS application and provides an excellent candidate for SERS analysis.
